# Call and be counted! Can we reliably estimate the number of callers in the indri's (*Indri indri*) song?

**DOI:** 10.1371/journal.pone.0201664

**Published:** 2018-08-03

**Authors:** Valeria Torti, Daria Valente, Chiara De Gregorio, Carlo Comazzi, Longondraza Miaretsoa, Jonah Ratsimbazafy, Cristina Giacoma, Marco Gamba

**Affiliations:** 1 Department of Life Sciences and Systems Biology, University of Torino, Torino, Italy; 2 Group d’Etude et de Recherche sur les Primates de Madagascar (GERP), Antananarivo, Madagascar; University of Sussex, UNITED KINGDOM

## Abstract

Estimating the number of animals participating in a choral display may contribute reliable information on animal population estimates, particularly when environmental or behavioral factors restrict the possibility of visual surveys. Difficulties in providing a reliable estimate of the number of singers in a chorus are many (e.g., background noise masking, overlap). In this work, we contributed data on the vocal chorusing of the indri lemurs (*Indri indri*), which emit howling cries, known as songs, uttered by two to five individuals. We examined whether we could estimate the number of emitters in a chorus by screening the fundamental frequency in the spectrograms and the total duration of the songs, and the reliability of those methods when compared to the real chorus size. The spectrographic investigation appears to provide reliable information on the number of animals participating in the chorusing only when this number is limited to two or three singers. We also found that the Acoustic Complexity Index positively correlated with the real chorus size, showing that an automated analysis of the chorus may provide information about the number of singers. We can state that song duration shows a correlation with the number of emitters but also shows a remarkable variation that remains unexplained. The accuracy of the estimates can reflect the high variability in chorus size, which could be affected by group composition, season and context. In future research, a greater focus on analyzing frequency change occurring during these collective vocal displays should improve our ability to detect individuals and allow a finer tuning of the acoustic methods that may serve for monitoring chorusing mammals.

## Introduction

Species living in social groups may communicate at long distance transmitting information through the use of loud vocal signals (e.g., carnivores [1]; primates [2,3]). Sometimes, the production of these signals is not limited to a single emitter but can involve the participation of several callers, resulting in a chorus display, that may or may not include all the animals in a group [4]. Although we do have limited knowledge of the ability of conspecifics in extracting information about the size of the group and its spatial position, behavioral observations suggested that these emissions play a significant role in spacing neighboring groups within an area and in regulating their social interactions [5,6].

Understanding the dynamics of choral singing is crucial in bioacoustics studies for many reasons [7,8], but possibly the most critical information is related to the potential of group chorusing in providing human listeners with information on the number of emitters. In fact, estimating the number of animals participating in a choral display is becoming essential in different research fields, from conservation biology to management censuses [9,10,11]. Understanding the number of animals in a chorus may also play a crucial role in the definition of reliable population estimates, particularly when the possibility of visual surveys is restricted by environmental or behavioral factors [12,13,14].

Investigating the vocal activity of a species instead of planning visual surveys is a highly cost-efficient technique [15] and is very popular in marine mammal studies [16,17,18]. Passive acoustic monitoring (PAM) is increasingly used in terrestrial and marine habitats [19,20,21]. One of its main benefits is that of minimizing the potential effect of the researcher on the behavior of the target animals. Of course, monitoring the presence of a species often represents only the first step before developing censuses that may provide researchers with estimates of the density and abundance of a particular species [22]. The potential advantage of using PAM recordings to count subjects is evident, but previous research has raised several methodological issues that led to use PAM as a complement to traditional recording techniques [21], especially in the case of terrestrial animals. Since acoustic signals do not propagate efficiently in air as they do in water, PAM only partially resolved the problems encountered with active monitoring for those species for which visual detection is largely limited [23]. However, the new wave of studies of ecoacoustics and the renovated interest in the study of soundscapes will surely contribute data for species and individual identification [24,25,26].

Although the spreading of new algorithms for automatic segmentation of the recordings and species identification are impressive [27,28], there is still a lack of information on the actual dynamics of group vocal displays and the potential for counting individuals. Studies on those terrestrial mammals which engage in complex choral displays represent a challenging natural example for individual recognition and may play a major role in estimating the relative or even absolute abundance of a species across an area [29,30]. In the case of several species displaying choruses (e.g. wolves [31,32]; jackals [33,34]; gibbons [35,36]), however, it has been impossible to validate the minimum number of emitters recognizable in a group because recordings could not be supplemented by traditional survey methods. The difficulties in providing a reliable estimate of the number of singers in a chorus are many, from background noise masking to individual behaviors that may affect acoustic propagation characteristics [37,38,39,40].

Canids were usually targeted as one of the most interesting cases of chorusing animals because their monitoring is one of the central issues in conservation biology [41,42]. The study of Passilongo and colleagues [43] showed that a spectral examination of the chorus howling allowed to estimate real versus bioacoustically predicted chorus size in a way far more precise and objective that field estimations by ear. The seminal work of Filibeck and colleagues [44] indicated that methods based on spectral sound decomposition could be effective in censuses of wolves via howls simulated by the howling technique [1]. In later studies, Root-Gutteridge [7,45], Passilongo [43,46], and colleagues found that fundamental frequency and amplitude variations could help identifying wolves with high accuracy. Passilongo and colleagues [43] showed that estimates generated by the screening of the spectrograms were closer to the real chorus size than the aural estimations of an expert operator. They also found that the reliability of chorus size estimates decreased with the increase of the real chorus size, especially when the latter exceeded four animals [43].

Thus, it is clear that direct observations of chorusing animals can vastly improve our understanding of the development, the dynamics and the structure of animal chorusing [47,7]. For this work, we contribute data on the vocal chorusing of a primate species, *Indri indri*. This species lives in socially monogamous family groups (2–6 individuals; [48]), usually consisting of the adult breeding couple and the offspring of up to four different generations. This lemur is mainly folivorous [49] and occupies and actively defends territories, whose extension varies according to the forest site [50]. Indris have a rich vocal repertoire (with eight different vocal types besides the song [51]) but they mainly rely on the emission of the so-called “songs” for regulating inter- and intra-group relationships [48]. indris are, in fact, among the so-called “singing primates” [52](like gibbons, tarsiers and titi-monkeys) and are the only lemur species producing songs, that can be heard up to 2 kilometers [53].

The indris' song consists of a long sequence of vocalizations that usually starts with a harsh emission ("roar"), followed by a series of slightly frequency modulated units ("long notes"). There is then a series of units organized in phrases with a descending frequency pattern (descending phrases) composed of 2–5 units [54]. Male and female indris within a group, including juveniles (aged up to one year, [55]), take part in a chorusing song, which lasts 40–250 s [51]. Previous studies have shown that male and female contributions to the song differ, both quantitatively and qualitatively, in the overall temporal structure of calling, repertoire size, and acoustic structure of the note types [56, 57]. Sex dimorphism is also present in the modulation of the frequency of vocal emissions, in the duration of note types and the rhythmic structure of a contribution [47]. The indris produce songs that differ in their acoustic structure between contexts [53, 54]. The songs may serve to inform the neighboring groups about the occupation of the territory (“advertisement songs”), to resolve territorial fights during a confrontation between neighbors (“territorial songs”), and have a cohesion function between group members (“cohesion songs”).

By visually detecting and observing every individual during the emission of their songs, we were able to apply the spectrographic methodology used in previous studies. We focused on estimating chorus size, and then we compared those estimates with the behavioral observations we did in the field. Direct observations not only allowed to assign each emission to an individual caller, but also improved our understanding of how many animals participated in the song, and how do they behave during singing. Because of these particular conditions, we were able, from time to time, to record videos that were used to describe how the indris emit their howling cries (e.g. S1 Movie).

Previous studies have shown that a weaker, but still useful information of group size could be derived from the total length of the choruses. Durbin [58] and Comazzi and colleagues [33] found that solo howls of the Asiatic wild dog (*Cuon alpinus*) and golden jackals (*Canis aureus*) were significantly shorter than chorus howls. Servin [59] showed that the average duration of chorus howling of the Mexican wolf (*Canis lupus baileyi*) varied significantly throughout the year, thus possibly related to a change in the number of vocalizers. Those evidences could explain why Harrington [60], who concentrated his sampling on a single season, found that the average duration of chorus howls by wolves did not vary with pack size or composition, while works on captive and wild animals showed that more wolves in a group might stimulate each other to longer howling [4,61,62]. The duration of an elicited chorus howl of free-ranging wolves (*Canis lupus*) significantly increased with group size [32]. Nowak and colleagues [32] reported that howls of single wolves or pairs lasted less than a minute (average: 34–40 s), whereas those of 5–7 wolves could reach nearly 4 minutes (average 67–95 s).

Palacios [63] examined the group howls of the Iberian wolf (*Canis lupus*) finding a correlation between chorus size and group size and between howl duration and group size. They showed that the number of wolves simultaneously vocalizing coincided for 73% of the howls with group size. The same authors found that the number of wolves vocalizing simultaneously and the length of the chorus increased with the number of wolves in a group. The correlation between group size and total duration of a chorus is far less investigated in nonhuman primates. Geissmann and Nijman [10] found a significant difference in the duration of female song bouts with one versus two participants.

At the light of the previous research, we examined whether we could estimate the number of indris emitting in a chorus using screening of the spectrograms and the reliability of this methods when compared to the real chorus size. We were expecting to find a correlation between chorus size and group size and between song duration and the number of vocalizing indris.

We could thus hypothesize that the spectrographic analyses of the indri’s choruses, based on the visual inspection of the fundamental frequency of each overlapping singer, could be predictive of the number of callers. We could also attend that the estimate we could generate with the spectrographic count would become less reliable [43] at the increase in the number of vocalizers. Moreover, we asked whether the duration of the song may be informative about the number of vocalizing animals or even of the size of the group. We could thus hypothesize that at the increase in the number of indri vocalizers we would observe an increase in chorus’ duration. Finally, we wanted to focus on whether song duration could provide cues about groups size, but we could not formalize a precise prediction for this last hypothesis because of the controversial data currently available from other species. We also tested whether the most common acoustic indices could provide information about the number of singers in a chorus.

## Methods

### Study areas, subjects and recordings

We studied 21 groups of indris living in three different areas of dense tropical forest in Madagascar (S1 Table): 9 groups in the Analamazaotra Reserve (Andasibe-Mantadia National Park, 18° 56’ S, 48° 25’ E), 3 groups in the Mitsinjo Station Forestière (18° 56’ S, 48° 24’ E), and 9 groups in the Maromizaha Forest (18° 56’ 49” S, 48° 27’ 53” E). We collected data in the field every year, from 2005 to 2016, for a total of 45 months. We observed one group per day from 06:00 am to 1:00 pm. We used natural marks to identify the individuals, and we had one observer per single indri during the observations to ensure we could correctly track each singer during the song.

Recordings were made using Sennheiser ME 66 and ME 67 and AKG CK 98 microphones. The microphone output signal was recorded using a solid-state digital audio recorder (Marantz PMD671, SoundDevices 702, Olympus S100 or Tascam DR-100MKII 24/96) at a sampling rate of 44.1 kHz. All utterances were recorded at a distance of maximum 10 m since all the study groups were habituated. We made all efforts to orient the microphone towards the vocalizing animal.

For this work, we selected songs emitted by two (duets) to five vocalizers (we use the term choruses for songs uttered by more than two indris) in 194 days of sampling (S1 Table). All the selected signals were advertisement songs [54].

### Acoustic and statistical analyses

**Fig 1:**
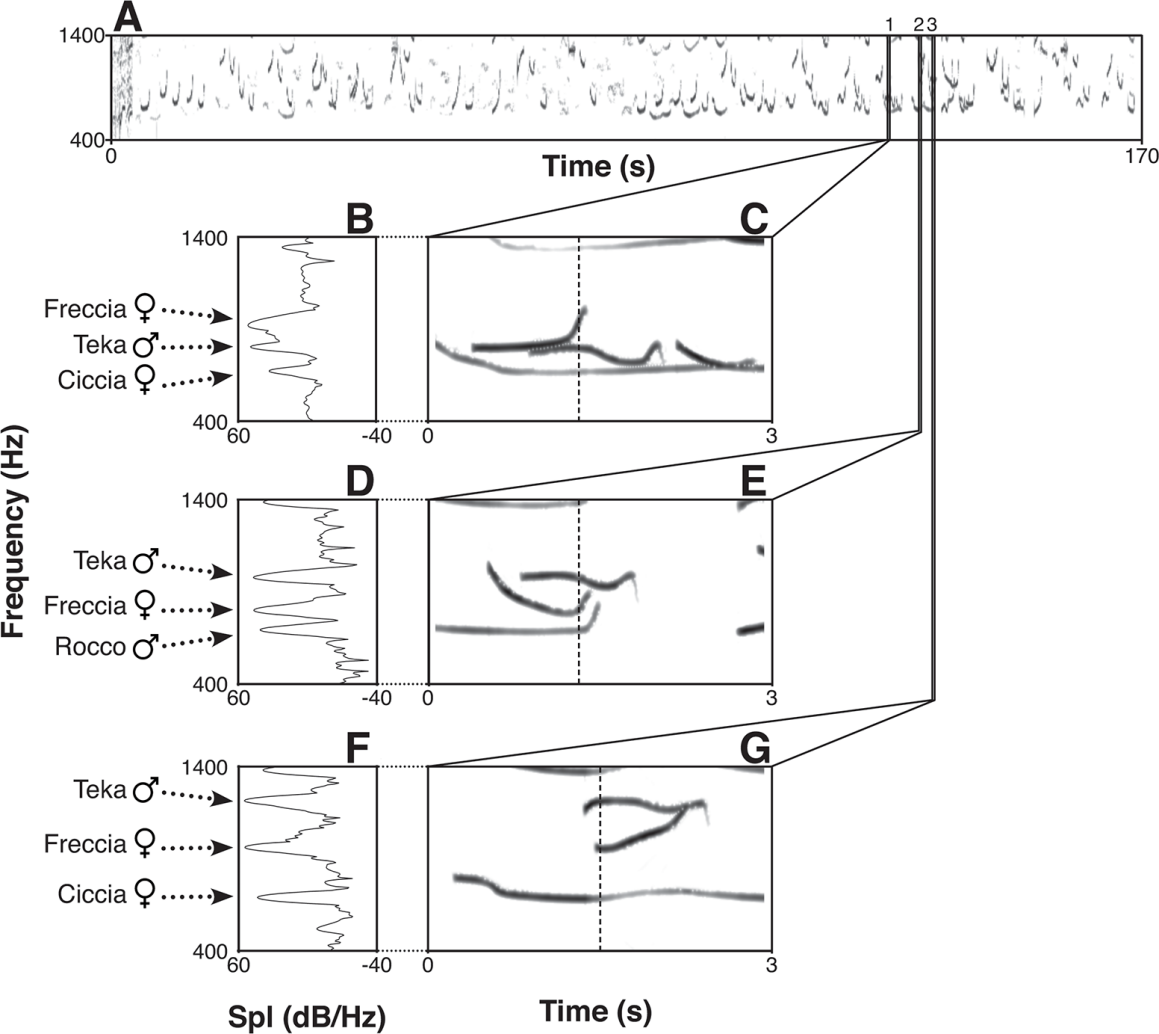
Spectrograms of the indris' song.

We analyzed a total of 258 songs (duets and choruses), which we edited using Praat 5.3.46 [64] and Boris 4.0.3 [65]. We used field notes and video recordings to assign every utterance to the correct emitter, as an individual profile (Fig 1). To extract information from the songs, we focused on the fundamental frequency (e.g the lowest frequency produced by the vibration of the vocal folds). We reported this information in a Praat textgrid [47].

To understand whether we could estimate the minimum number of callers in a song, we carefully screened the spectrogram (e.g. the visual representation of the spectrum of frequencies of a sound in relation to time) of each song in Praat, focusing on a frequency range of 0 to 3000 Hz and using a window length of 0.025 s. Following a methodology similar to those utilized in the works of Filibeck et al. [44], Passilongo et al. [43], and Comazzi et al. [33], we visually identified the portions of the chorus in which we could recognize multiple vocalizers, by identifying overlapping notes (each with its fundamental frequency profile) and counting the number of overlapping fundamental frequencies. Given the fact that we attributed each vocalization in the song to its respective vocalizer, the maximum value of simultaneous emitters was taken as the estimate of the minimum number of participants for that chorus (Predicted Chorus Size, CSp, following Passilongo et al. [43]). We used our field notes and video recordings to track the real number of animals singing in a particular chorus (CS_r_) and the group size of the group in that season (GS). We used a Praat script to extract and save the total duration of the chorus in a text file, which could be then exported to a comma-separated file [66,67] or used in R (The R Foundation for Statistical Computing 2017; version 3.3.3).

We investigated to what extent we could spectrographically predict the exact number of callers in a song by calculating the percentage of songs in which CSp and CSr coincided. We did this calculation by grouping data according to CSr. We then calculated four acoustic diversity indices for each indri’s song (seewave [68] and soundecology packages [69] in R): 1) the acoustic complexity index, ACI [70]; 2) the acoustic richness index, AR [71]; 3) the acoustic diversity index, ADI [72] and the Acoustic Entropy Index, H [73]. The four indexes (see for details [74, 75]) are applied here to understand whether they could inform about the numerosity of singers in a chorus. We considered the frequency range between 150 Hz and 10000 Hz (comprising the fundamental frequency and the visible harmonics) and used a Fast Fourier Transform window of 512 samples.

We ran the Spearman correlation test in R (The R Foundation for Statistical Computing 2017; version 3.3.3) to understand whether there was a correlation between the duration of the choral songs and CSp, CSr, and GS. We also run the Spearman correlation test to reveal whether there was a significant correlation between each index (ACI, AR, ADI, H) and the real number of singers (CSr) in the choruses. We also calculated the confidence interval for each set of data by using the spearman.ci function (package RVAideMemoire 0.9–68; [76]). We visualized all our analyses and calculations using the package ggplot2 2.2.1 [77].

We ran four separate linear mixed-effects models (LMM, lme4 package in R) for song duration and CSp, with the real number of animals singing in a particular chorus (CSr) and the group size of the group in that season (GS), entered as the response variables. We tested for the distribution of both the responses and the fixed variables. We log-transformed the data when the data were not normally distributed.

The two LMM models we used to investigate the variation in the real number of singers (CSr) included the predicted chorus size (CSp) and the song duration alternatively, as fixed factors. The two models in which the group size of the group in that season (GS) was the response variable included the predicted chorus size (CSp) and the song duration alternatively, as fixed factors. We entered group identity as a random factor in all the four models. We finally ran a chi-square test in R to define which variables turned out to be predictive.

We then run four separate linear mixed-effects models (LMM) to investigate the variation of the four different acoustic indexes (ACI, AR, ADI, H), adding the real number of singers (CSr) in the choruses as a fixed factor. We entered group identity as a random factor in all the four models.

For every LMM model, we verified the assumptions that the residuals were normally distributed and homogeneous by looking at a quantile-quantile plot and the distribution of the residuals plotted against the fitted values with a specific function written by R. Mundry (Max Planck Institute, Germany). To test the significance of each full model [78] we compared it against a null model comprising the random factor (group identity) exclusively, by using a likelihood ratio test (Anova with argument test “Chisq” [79]). Then, we calculated the P values for the predictors based on likelihood ratio tests between the full and the respective null model. We, finally, calculated conditional R-squared (R^2^_C_) measures for each LMM model, providing an absolute value for the goodness-of-fit of each model [80].

A spectrogram (A) of a complete song generated using Praat (time step: 20 ms). We enlarged three overlap occurrences between the singers (C, E, and G) and presented the spectra (B, D, and F) calculated at the point indicated by the dashed line in each spectrogram. The occurrence of different fundamental frequencies (f0s) and harmonic structures at the same time allows indicating the emission of different animals. The arrows indicate the name and sex of the vocalizing individual and the respective spectral peak. Notice the variation of the Sound Pressure Level (SPL) of an individual across the different spectra. Other peaks may correspond background noise and harmonics.

## Results

### Predicted chorus size vs real chorus size

**Fig 2:**
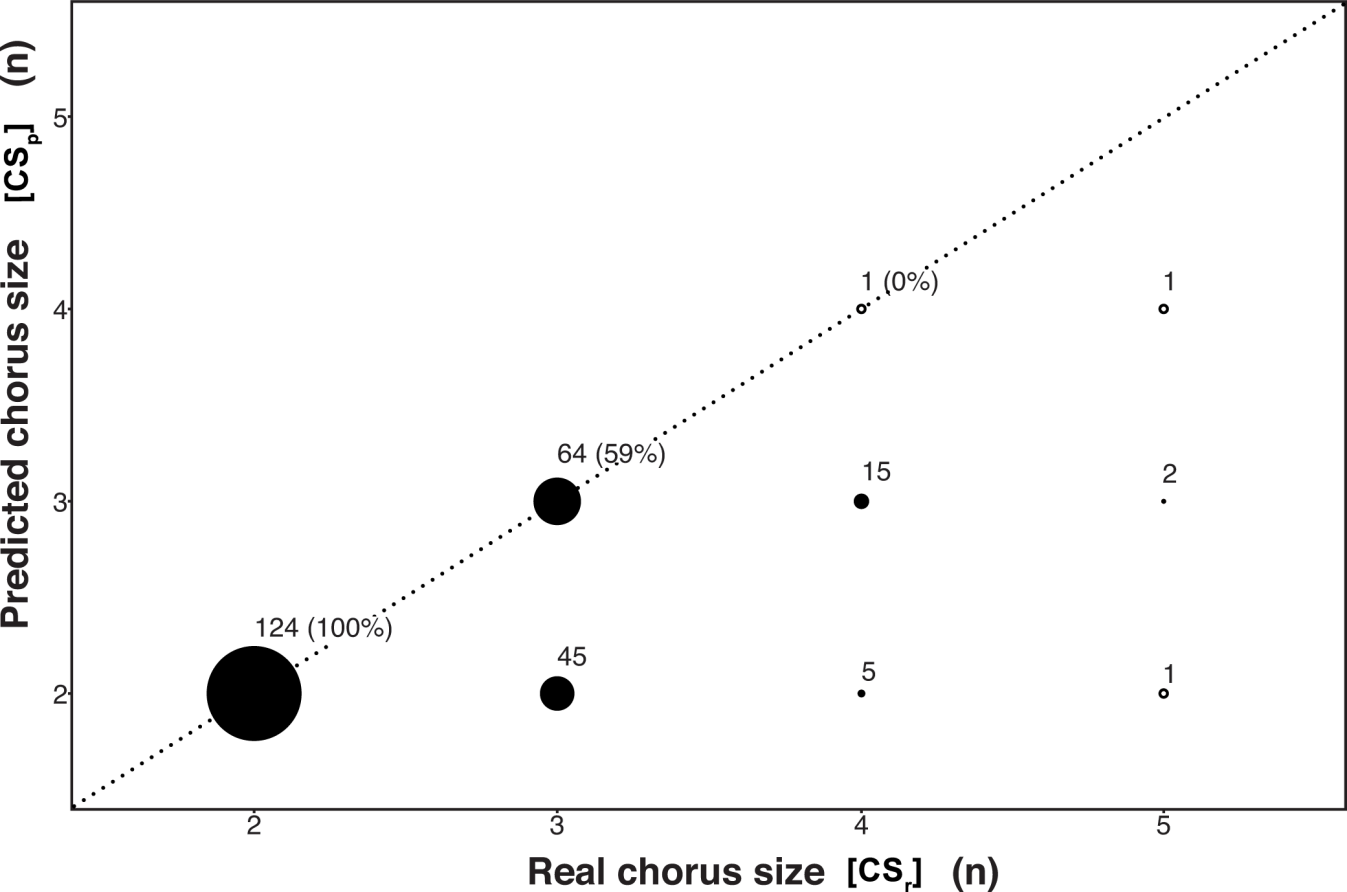
Spectrographically predicted versus real chorus size in the indris.

We compared CS_p_ with CS_r_ to understand to what extent the spectrographic investigation could provide accurate estimates of the number of chorusing animals (see S2 Table for differences between groups). It has been interesting to notice that only in 165 cases all the group members actively sung in the chorus. In 74 recordings, more than 50% took part to the song, and in 19 songs we observed 50% or less of the group participating to the chorus. The estimated number of emitters ranged from two (N = 175) to four (N = 2), while the real chorus size ranged from two (N = 124) to five (N = 4; Fig 2). In all the songs emitted by pairs (N = 124), we could indicate two callers from the spectrogram screening. Thus, for the groups where we had a pair only, real group size and estimated group size coincided. Songs emitted by three indris (N = 109) showed the potential to indicate all the emitters only in 64 cases (59%). The remaining 45 songs allowed indicating only two emitters. We had 21 songs in which four singers coordinated their emissions. In five of them (24%) we could indicate only two emitters from the spectrograms (Fig 2). In 15 of the remaining songs (71%), we could indicate three singers, and only one song allowed indicating from the spectrogram the real chorus size of four emitters (Fig 2). Four songs, in which we observed five singers participating, revealed an estimate of 2, 3 and four emitters (Fig 2).

Scatter plot of Spectrographically predicted (CS_p_) versus Real chorus size (CS_r_) in the indris. The radius of each point is proportional to the number of cases, which is reported with the percentage of correct estimates. A circled white dot denotes N = 1. The diagonal indicates the accurate predictions.

### Duration vs real chorus size

**Fig 3:**
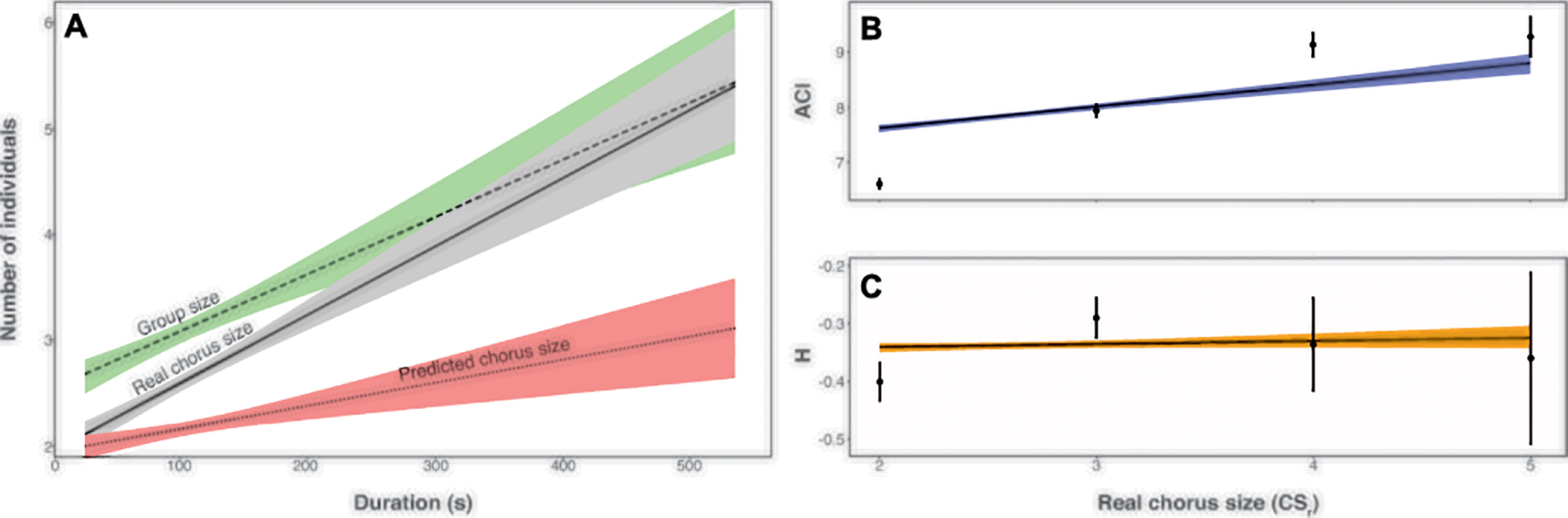
Indri’s song duration. The indris’ song duration is (A) presented as a function of Group size (dashed line), Real chorus size (solid line), and Predicted chorus size (dotted line). The Real chorus size is presented as a function of (B) the Acoustic complexity index (ACI) and (C) the Acoustic entropy index (H). Each regression line is represented with the associated 95% confidence interval range (shades). Error bars indicate standard errors.

The duration of indri choruses ranged between 25.32 s and 533.79 s. We found a positive correlation between the real number of singers and the overall duration of the song (Spearman correlation test; N = 258, p < 0.001; rho = 0.601, Fig 3A) as it has already been reported by Gamba and colleagues [47]. The correlation between the estimated number of singers and the total duration was also positive and significant (Spearman correlation test; N = 258, p < 0.001; rho = 0.313, Fig 3). When we considered duration in the light of real chorus size, we found that songs emitted by pairs ranged between 25.320 s and 191.960 s (76.533 ± 29.649, N = 124). Songs emitted by three indris lasted from 42.300 s to 533.790 s (119.576 ± 59.067, N = 109), while those given 4 or 5 lemurs showed a minimum duration of 91.510 s and a maximum of 404.900 s (183.531 ± 73.980, N = 25). It may be of interest to notice that also group size (calculated on the potential singers of the group, e.g., adults and subadults according to Pollock 1986) positively correlated with song duration (Spearman correlation test; N = 258, p < 0.001; rho = 0.418, Fig 3).

### Comparison between models

We first estimated to what extent the real number of animals singing in a particular chorus (CS_r_) could be predicted by CS_p_ and by song duration. CS_p_ and song duration significantly explained the CS_r_ variability (Table 1). CS_p_ values (R^2^_C_ = 0.519) and song duration (R^2^_C_ = 0.522) explained the same percentage of variation in CS_r_ (52%). The two models did not differ (df = 4; p = 1.000).

**Table 1:** Results of the LMM full models for CS_r_ and GS.

**Real number of animals singing in a particular chorus (CS**_**r**_**)** CS_p_ model: full vs. null; chisq = 101.0795, df = 1, p < 0.001 Song duration model: full vs. null; chisq = 88.877, df = 1, p < 0.001				
	Estimate	SE	t	P
*(Intercept)*	0.362	0.057	6.381	^a^
*CS*_*p*_	0.684	0.060	11.313	<0.001
*(Intercept)*	-0.271	0.119	-2.280	^a^
*song duration*	0.265	0.026	10.377	<0.001
**Group size of the group in that season (GS)** CS_p_ model: full vs. null; chisq = 10.860, df = 1, p = 0.001 Song duration model: full vs. null; chisq = 18.888, df = 1, p < 0.001				
	Estimate	SE	t	P
*(Intercept)*	0.972	0.067	14.574	^a^
*CS*_*p*_	0.160	0.048	3.337	0.001
*(Intercept)*	0.718	0.102	7.032	^a^
*song duration*	0.085	0.019	4.428	<0.001

Influence of the fixed factors on the real number of animals singing in a particular chorus (CS_r_) and on group size (GS).

^a^Not shown as not having a meaningful interpretation.

We then tested whether group size (GS) could also be predicted by CS_p_ and by song duration. The GLM full models (Table 1) returned a conditional R-squared of R^2^_C_ = 0.788 and R^2^_C_ = 0.795 respectively. Both predictors significantly explained GS variability (Table 1). When compared, the two models were significantly different (Chi square; df = 4; p < 0.001). We also observed that song duration better predicts group size (GS) if compared to CS_p_.

### Bioacoustics diversity vs real chorus size

We found a positive correlation between the acoustic complexity index (ACI) and the real number of singers in a chorus (Spearman correlation test; N = 258, p < 0.001; rho = 0.578). We did not find a correlation between the real number of singers in a chorus and the ADI (Spearman correlation test; N = 258, p = 0.454; rho = -0.047), the AR (Spearman correlation test; N = 258, p = 0.955; rho = -0.004), and the H (Spearman correlation test; N = 258, p = 0.113; rho = 0.098) indices. The real chorus size had a significant effect on the acoustic complexity index (ACI), and the acoustic entropy index (H, Table 2). The LMM models did not show this significant relationship for the other two indexes (ADI and AR).

**Table 2:** Results of the LMM full models for the acoustic indices.

**Acoustic Diversity Index (ADI)** full vs. null; chisq = 0.078, df = 1, p = 0.780				
	Estimate	SE	t	P
*(Intercept)*	0.736	0.015	47.646	^a^
*CS*_*r*_	-0.004	0.015	-0.279	0.780
**Acoustic Complexity Index (ACI)** full vs. null; chisq = 87.559, df = 1, p < 0.001				
	Estimate	SE	t	P
*(Intercept)*	6.829	0.105	64.900	^a^
*CS*_*r*_	1.109	0.106	10.473	<0.001
**Acoustic Entropy Index (H)** full vs. null; chisq = 5.024, df = 1, p < 0.001				
	Estimate	SE	t	P
*(Intercept)*	0.692	0.008	82.219	^a^
*CS*_*r*_	0.018	0.008	2.253	0.025
**Acoustic Richness Index (AR)** full vs. null; chisq = 0.180, df = 1, p = 0.671				
	Estimate	SE	t	P
*(Intercept)*	0.302	0.076	3.958	^a^
*CS*_*r*_	-0.302	0.076	-0.426	0.671

Influences of the real number of animals singing in a particular chorus (CS_r_) on the different acoustic diversity indexes.

^a^Not shown as not having a meaningful interpretation.

By direct comparison of the R-squared values for the acoustic diversity indexes, we observed that the real chorus size better predicts the acoustic complexity index variation (R^2^_C_ = 0.386) than the acoustic entropy index variation (R^2^_C_ = 0.219) (Fig 3B and 3C).

## Discussion

In this paper, we evaluated whether chorusing displays may provide reliable insights into the number of individuals participating in the chorus, contributing new data from direct observation of singing lemurs. The spectrographic investigation provided reliable information on the number of animals engaging in the chorusing only when this number is limited to two or three singers. This outcome supports a systematic underestimation of the number of callers, which has been already indicated in a previous study [43]. Our findings are congruent with those presented by Passilongo and colleagues [43] and Comazzi and colleagues [33] on wolves and jackals. Even if the spectrographic investigation is useful for predicting the real number of singers, the method allowed estimating the actual chorus size correctly only for small chorus sizes. At the increase in the number of emitters, the reliability of the estimate decreased. Thus, whether minimum counts of the individuals in a group are useful, it must be considered that they may not reflect the actual chorus size.

In *Indri indri* solo singing is rare [47] and is mainly observed in dispersing individuals (VT personal observations), playing a significant role in mate attraction and new pairs’ settling [53,54,47]. Songs are frequently emitted by all the singing individuals in a family group, even if we observed that all group members participate to the song more frequently during territorial fights than in advertisement songs [54], in agreement with data of Bonadonna and colleagues [48]. Besides, despite high levels of overlapping between singers’, indris show significant non-overlapping rates in the song emission. We previously found that an indri dominance status (e.g. reproductive vs non-reproductive members) plays a crucial role in the amount of overlap in the song [47]. Animals showed overlapping avoidance in between dominants and non-dominants (which are often sub-adults and offspring in our sampling) and more frequent overlapping between the adult reproductive couple. Thus, the underestimation of the number of callers could be directly influenced by the low levels of overlapping between the breeding couple and more than one non-dominant individual. The underestimation for more than three singers can be partially explained by the fact that, when more than three animals are singing in a chorus, they may avoid or show small overlap [47]. We observed, in fact, that at the increase in the number of singers in a chorus the amount of co-singing between two individuals significantly decreased [47].

We tested whether the most common acoustic indices could inform about the number of indris vocalizing in a song. We found the acoustic complexity index and acoustic entropy index correlated with the observed chorus size. These results are particularly interesting because acoustic indexes are more often used in the field of ecology, but their application to investigate animal behavior is rare. The current result confirms that ACI and H would be suitable for further evaluation of animal chorusing. The number of indris participating to the song had no significant influence on the other indices we tested.

The extent to which these estimates can reflect the actual group size in the indris is still difficult to understand, as the participation of all members of the group to the chorus appears variable. The relation between chorus size and group size may be affected by group composition (e.g.; age and sex of the group members), season and context of emission.

A different conclusion can be taken when considering whether the number of singers may affect song duration and to what extent song duration can inform about group size. Overall, we found evidence of the higher predictive power of total duration if compared to the spectrographic investigation. Song duration appeared to better explain the variability of both the real chorus size and the group size if compared to spectrographically predicted chorus size. Describing the temporal properties of the indris’ singing, we found that the individuals tend to overlap more in duets than in choruses. While adults tend to overlap, in fact, sub-adults are more likely to avoid co-signing. Consequently, preventing overlap between individual contributions could result in increasing the total duration of the songs. Song duration could better predict the number of singers, even if this effect could be masked by several factors (e.g. season, sex of the co-emitter, age, etc.). Both song duration and CSp, in fact, show the same power in predicting the real number of singers. The reliability of both methods tends to decrease at the increase in the number of singers in a chorus. For those reasons, the song duration model shows a remarkable variation that remains unexplained.

Finally, we could confirm that song duration better predicts both the real number of animals singing in a particular chorus and group size (GS) if compared to the analysis of the fundamental frequency profiles. At least for the advertisement songs, total duration appears to a reliable of real chorus size.

In future research, a greater focus on analyzing frequency variation occurring during these collective vocal displays should improve our ability to detect individuals [81] and allow finer tuning of the acoustic methods that may serve for monitoring chorusing mammals.

## Supporting information

S1 Movie Indri chorus. An extract from a reproductive pair’s duet.
(MP4)

S1 Table Summary of the dataset. Summary of the dataset with group ID, site, year of the recording, individual, sex, number of singers (mean+sd), group size (mean+sd) and number of days in which the songs were recorded. * symbol denotes that the individual is an adult (aged more than 6 years) at the time of the recording (column Year); R symbol indicates that the individual is member of the reproductive couple; R2 symbol denotes that the individual has been involved in a takeover and is the new reproductive member of the couple. Numbers in round brackets represent the year in which an individual moved away from the group. When we have evidences of the death of an animal we inserted the year with the †.
(DOCX)

S2 Table Results of the Tukey’s HSD (honestly significant difference) applied to the group size during our study. The groups are listed in order of ascending harmonic means (mean±se). Subset 1: p = 0.122; Subset 2: p = 0.060.
(DOCX)
