# Supplementary material for: Call and be counted! Can we reliably estimate the number of callers in the indri's (Indri indri) song?
Source: PLoS One. 2018 Aug 3;13(8):e0201664. doi: 10.1371/journal.pone.0201664 (PMC6075759; doi:10.1371/journal.pone.0201664)
Supplement: S1 Table — Summary of the dataset with group ID, site, year of the recording, individual, sex, number of singers (mean+sd), group size (mean+sd) and number of days in which the songs were recorded. * symbol denotes that the individual is an adult (aged more than 6 years) at the time of the recording (column Year); R symbol indicates that the individual is member of the reproductive couple; R2 symbol denotes that the individual has been involved in a takeover and is the new reproductive member of the couple. Numbers in round brackets represent the year in which an individual moved away from the group. When we have evidences of the death of an animal we inserted the year with the †. (DOCX) [file pone.0201664.s002.docx]

**S1 Table. Summary of the dataset.**

| ***Group ID*** | ***Site*** | ***Year*** | ***N songs*** | ***Individual*** | ***Sex*** | ***N° singers (mean+SD)*** | ***Group size (mean+SD)*** | ***N° days of recordings*** |
| --- | --- | --- | --- | --- | --- | --- | --- | --- |
| 1MZ | Maromizaha | 2011 2012 2016 | 46 | Jery*^,R^ | M | 2,70+0,55 | 3,00+1,00 | 29 |
|  |  |  |  | Bevolo*^,R^ | F |  |  |  |
|  |  |  |  | Fotsy* (2013) | M |  |  |  |
|  |  |  |  | Maintso (2014) | F |  |  |  |
|  |  |  |  | Berthe (2014) | F |  |  |  |
| 2MZ | Maromizaha | 2011 2012 2016 | 21 | Max*^,R^ | M | 2,00+0,00 | 2,00+0,00 | 13 |
|  |  |  |  | Soa*^,R^ | F |  |  |  |
| 3MZ | Maromizaha | 2011 2012 2015 2016 | 42 | Ratsy*^,R^ (†2015) | M | 2,64+0,62 | 3,30+1,00 | 31 |
|  |  |  |  | Mahagaga*^,R2^ | M |  |  |  |
|  |  |  |  | Mena*^,R^ | F |  |  |  |
|  |  |  |  | Zandry (2015) | F |  |  |  |
|  |  |  |  | Tonga | F |  |  |  |
|  |  |  |  | Faly | M |  |  |  |
| 4MZ | Maromizaha | 2015 2016 | 5 | Koto*^,R^ | M | 3,60+0,55 | 4,00+0,00 | 5 |
|  |  |  |  | Eva*^,R^ | F |  |  |  |
|  |  |  |  | Hendry* | M |  |  |  |
|  |  |  |  | Gibet | M |  |  |  |
| 5MZ | Maromizaha | 2016 | 2 | Graham*^,R^ | M | 2,00+0,00 | 4,00+0,00 | 2 |
|  |  |  |  | Fern*^,R^ | F |  |  |  |
|  |  |  |  | Sonny Bill | F |  |  |  |
|  |  |  |  | All Black | M |  |  |  |
| 6MZ | Maromizaha | 2016 | 5 | Zokibe*^,R^ | M | 2,00+0,00 | 3,00+0,00 | 4 |
|  |  |  |  | Befotsy*^,R^ | F |  |  |  |
|  |  |  |  | Hira | M |  |  |  |
| 8MZ | Maromizaha | 2016 2017 | 6 | Jonah*^,R^ | M | 3,50+1,38 | 4,00+1,00 | 5 |
|  |  |  |  | Bemasoandro*^,R^ | F |  |  |  |
|  |  |  |  | Cesare* | M |  |  |  |
|  |  |  |  | Mika | F |  |  |  |
|  |  |  |  | Zafy | M |  |  |  |
| 9MZ | Maromizaha | 2016 | 4 | Emilio*^,R^ | M | 2,50+0,58 | 3,00+0,00 | 4 |
|  |  |  |  | Sissie*^,R^ | F |  |  |  |
|  |  |  |  | Dosy | F |  |  |  |
| 10MZ | Maromizaha | 2016 | 2 | Tia*^,R^ | M | 2,00+0,00 | 3,00+0,00 | 2 |
|  |  |  |  | Joeline*^,R^ | F |  |  |  |
|  |  |  |  | Voandalana | ? |  |  |  |
| 1R | Analamazaotra | 2005 2007 2008 | 17 | Curvo*^,R^ | M | 2,76+0,44 | 3,70+0,60 | 13 |
|  |  |  |  | Tozza*^,R^ | F |  |  |  |
|  |  |  |  | Cleo | M |  |  |  |
|  |  |  |  | Norma (2008) | F |  |  |  |
|  |  |  |  | Panda | ? |  |  |  |
| 2R | Analamazaotra | 2005 2007 2008 | 11 | Rocco*^,R^ | M | 3,64+0,92 | 4,70+0,60 | 10 |
|  |  |  |  | Freccia*^,R^ | F |  |  |  |
|  |  |  |  | Ciccia | F |  |  |  |
|  |  |  |  | Teboka*^,R2^ | M |  |  |  |
|  |  |  |  | Stringo (†2006) | M |  |  |  |
|  |  |  |  | Forchette (†2007) | F |  |  |  |
|  |  |  |  | Teka | M |  |  |  |
| 3R | Analamazaotra | 2005 2006 2007 2008 | 24 | Fano*^,R^ (2007) | M | 3,04+0,69 | 4,30+0,50 | 20 |
|  |  |  |  | Mamatin*^,R^ | F |  |  |  |
|  |  |  |  | Tete Dure*^,R2^ | M |  |  |  |
|  |  |  |  | Chef* | M |  |  |  |
|  |  |  |  | Zaza | M |  |  |  |
|  |  |  |  | Lanto | M |  |  |  |
| 5R | Analamazaotra | 2005 2006 2007 2008 | 15 | Ranga*^,R^ | M | 2,00+0,00 | 2,00+0,00 | 13 |
|  |  |  |  | Sola*^,R^ (2007) | F |  |  |  |
|  |  |  |  | Clara*^,R2^ | F |  |  |  |
| 6R | Analamazaotra | 2005 2007 2008 | 12 | Plateau*^,R^ | M | 2,67+0,49 | 3,70+0,60 | 6 |
|  |  |  |  | Nbola*^,R^ | F |  |  |  |
|  |  |  |  | Ninja | M |  |  |  |
|  |  |  |  | Cammela | F |  |  |  |
| XR | Analamazaotra | 2005 2007 2008 | 10 | Fumo*^,R^ | M | 2,00+0,00 | 2,00+0,00 | 8 |
|  |  |  |  | Vola Mena*^,R^ | F |  |  |  |
| YR | Analamazaotra | 2007 | 2 | Bekibo*^,R^ | M | 3,00+0,00 | 3,00+0,00 | 2 |
|  |  |  |  | Celeste*^,R^ | F |  |  |  |
|  |  |  |  | Armando | M |  |  |  |
| 1M | Mantadia | 2006 2007 | 4 | Maquillage*^,R^ | M | 2,75+0,50 | 3,50+0,70 | 3 |
|  |  |  |  | Cristina*^,R^ | F |  |  |  |
|  |  |  |  | Faralahy | M |  |  |  |
|  |  |  |  | Mère Noel | F |  |  |  |
| 4M | Mantadia | 2007 | 3 | Jobby*^,R^ | M | 2,00+0,00 | 2,00+0,00 | 3 |
|  |  |  |  | Chiara*^,R^ | F |  |  |  |
| ASF | Mitsinjo SR | 2005 2008 | 10 | Rambo*^,R^ | M | 3,10+0,57 | 3,50+0,70 | 9 |
|  |  |  |  | Maya*^,R^ | F |  |  |  |
|  |  |  |  | Lion* (2008) | M |  |  |  |
|  |  |  |  | Jule (2006) | M |  |  |  |
|  |  |  |  | Sabotsy | M |  |  |  |
|  |  |  |  | Talata | M |  |  |  |
| WSF | Mitsinjo SR | 2005 2008 | 6 | Rasta*^,R^ | M | 2,00+0,00 | 2,00+0,00 | 3 |
|  |  |  |  | Tina*^,R^ | F |  |  |  |
|  |  |  |  | Mauro | M |  |  |  |
| YSF | Mitsinjo SR | 2005 2007 2008 | 11 | Panza*^,R^ | M | 2,45+0,52 | 3,7+0,60 | 9 |
|  |  |  |  | Lisa*^,R^ | F |  |  |  |
|  |  |  |  | Diadème | F |  |  |  |
|  |  |  |  | Miss Scarlet | F |  |  |  |
|  |  |  |  | Christo | F |  |  |  |

Summary of the dataset with group ID, site, year of the recording, individual, sex, number of singers (mean+sd), group size (mean+sd) and number of days in which the songs were recorded. * symbol denotes that the individual is an adult (aged more than 6 years) at the time of the recording (column Year); ^R^ symbol indicates that the individual is member of the reproductive couple; ^R2^ symbol denotes that the individual has been involved in a takeover and is the new reproductive member of the couple. Numbers in round brackets represent the year in which an individual moved away from the group. When we have evidences of the death of an animal we inserted the year with the †.
