# Supplementary material for: Call and be counted! Can we reliably estimate the number of callers in the indri's (Indri indri) song?
Source: PLoS One. 2018 Aug 3;13(8):e0201664. doi: 10.1371/journal.pone.0201664 (PMC6075759; doi:10.1371/journal.pone.0201664)
Supplement: S2 Table — The groups are listed in order of ascending harmonic means (mean±se). Subset 1: p = 0.122; Subset 2: p = 0.060. (DOCX) [file pone.0201664.s003.docx]

**S2 Table**. **Results of the Tukey’s HSD (honestly significant difference) applied to the group size during our study.**

| **group** | **Subset 1** | **Subset 2** |
| --- | --- | --- |
| 10MZ | 2.00+0.00 |  |
| 2MZ | 2.00+0.00 |  |
| 4M | 2.00+0.00 |  |
| 5MZ | 2.00+0.00 |  |
| 5R | 2.00+0.00 |  |
| 6MZ | 2.00+0.00 |  |
| WSF | 2.00+0.00 |  |
| XR | 2.00+0.00 |  |
| YSF | 2.45+0.16 | 2.45+0.16 |
| 9MZ | 2.50+0.29 | 2.50+0.29 |
| 3MZ | 2.64+0.09 | 2.64+0.09 |
| 6R | 2.67+0.14 | 2.67+0.14 |
| 1MZ | 2.70+0.08 | 2.70+0.08 |
| 1M | 2.75+0.25 | 2.75+0.25 |
| 1R | 2.76+0.11 | 2.76+0.11 |
| XR | 3.00+0.00 | 3.00+0.00 |
| 3R | 3.04+0.14 | 3.04+0.14 |
| ASF | 3.10+0.18 | 3.10+0.18 |
| 8MZ |  | 3.50+0.56 |
| 4MZ |  | 3.60+0.24 |
| 2R |  | 3.64+0.28 |

The groups are listed in order of ascending harmonic means (mean+se). Subset 1: p = 0.122; Subset 2: p = 0.060.
